# Evolution and Association Analysis of *Ghd7* in Rice

**DOI:** 10.1371/journal.pone.0034021

**Published:** 2012-05-30

**Authors:** Li Lu, Wenhao Yan, Weiya Xue, Di Shao, Yongzhong Xing

**Affiliations:** National Key Laboratory of Crop Genetic Improvement and National Center of Plant Gene Research (Wuhan), Huazhong Agricultural University, Wuhan, China; University of Lausanne, Switzerland

## Abstract

Plant height, heading date, and yield are the main targets for rice genetic improvement. *Ghd7* is a pleiotropic gene that controls the aforementioned traits simultaneously. In this study, a rice germplasm collection of 104 accessions (*Oryza sativa*) and 3 wild rice varieties (*O.rufipogon*) was used to analyze the evolution and association of *Ghd7* with plant height, heading date, and yield. Among the 104 accessions, 76 single nucleotide polymorphisms (SNPs) and six insertions and deletions were found within a 3932-bp DNA fragment of *Ghd7*. A higher pairwise π and θ in the promoter indicated a highly diversified promoter of *Ghd7*. Sixteen haplotypes and 8 types of Ghd7 protein were detected. SNP changes between haplotypes indicated that *Ghd7* evolved from two distinct ancestral gene pools, and independent domestication processes were detected in *indica* and *japonica* varietals respectively. In addition to the previously reported premature stop mutation in the first exon of *Ghd7*, which caused phenotypic changes of multiple traits, we found another functional C/T mutation (SNP S_555) by structure-based association analysis. SNP S_555 is located in the promoter and was related to plant height probably by altering gene expression. Moreover, another seven SNP mutations in complete linkage were found to be associated with the number of spikelets per panicle, regardless of the photoperiod. These associations provide the potential for flexibility of *Ghd7* application in rice breeding programs.

## Introduction

Extensive archaeological evidence indicates that rice was first domesticated along the middle and lower Yangtze River corridors in South China about 8000 years ago [1], [2]. In this domestication process, two genetically distinct *Oryza sativa* subspecies, *indica* and *japonica*, were formed. These two rice subspecies can be distinguished by both DNA markers and morphologic characteristics [3]. Genotyping by Londo *et al.* [4] showed that *indica* and *japonica* subspecies arose from genetically distinct gene pools within a common wild rice ancestor (*Oryza rufipogon*) in South Himalaya and Southern China respectively. The domestication of the two subspecies occurred independently in different ecological and geographical environments. In both subspecies human selection has maintained the introgressions containing the most agriculturally valuable alleles [5]. Besides the two subspecies (*indica* and *japonica*), a deeper population structure has been defined by previous researchers, subdividing them into five genetically distinct subpopulations: *indica*, *aus*, *temperate japonica*, *tropical japonica* and *aromatic* [6].

Recently, candidate gene association analysis has been used to trace the origin of agronomically important alleles and to explore the domestication process of cultivated rice. For example, a single nucleotide polymorphism (SNP) in the predicted DNA-binding domain of the grain-shattering gene, *Sh4*, reduced the degree of shattering, resulting in a critical improvement in the rice harvest [7]. The *Sh4* mutation is prevalent in all cultivated rice varieties but is absent in wild rice, implying an essential role for this allele during rice domestication[7]–[9]. In a segregating population resulting from a cross between *japonica* and *indica*, a single nucleotide change located 12 kb upstream of another shattering gene in rice, *qSH1,* was found to decrease the expression of *qSH1* and consequently reduced grain shattering. In this case, the mutation was limited to the *temperate japonica* subpopulation without dissemination, indicating that independent domestication processes of subpopulations existed during the evolution of rice [10]. Similarly, a *temperate japonica* subpopulation specific allele showing limited dissemination was established for the *waxy* gene where an intron splice donor site mutation is responsible for the absence of amylose in the rice endosperm starch [11]. The *Rc* gene responsible for red seed color in wild rice showed two types of domestication selection sweeps. In one type, a frame-shift deletion within *Rc* was found in 97.9% of white rice varieties. This deletion originated in *japonica* and then was transferred into *indica* [12], [13]. In another case, a natural allelic variant of *Rc* called *Rc-s*, which is present in <3% of white accessions and shows limited dissemination, has a stop codon that originated in *indica* [12], [13]. The association analysis of the cloned domestication genes in a population has offered an opportunity to better understand the gene function and has provided insight into the evolutionary history of rice.

Yield is one of the most important traits that has been closely examined in the history of domestication; however, it is a complex trait determined by three component traits: number of panicles, number of grains per panicle, and grain weight, all of which are quantitative traits controlled by quantitative trait loci and influenced by environmental conditions [14]. Although this quantitative trait is under the control of numerous genetic components, a few key discrete genetic loci appear to be involved in yield increase during rice domestication [15]. Recently, a number of genes that control rice yield and adaptation have been identified through map-based cloning. For example, an amino acid substitution in PROG1 protein changed the rice plant architecture from the prostrate growth of wild rice to the erect growth habit of domesticated rice, concurrently resulting in increased grain yield [16], [17]. In addition, haplotype analysis revealed that this *PROG1* allele was fixed during rice domestication since identical alleles were detected in all accessions of *O. sativa* [16], [17].

Heading date (HD) is also an important determinant of rice yield. Rice is a short-day plant, with the distribution of the ancestral species located in the tropics. The domesticated rice growing area was extended to the Northern latitudes by selecting accessions with appropriate heading dates [18]. Flowering in rice is controlled by *Hd3a*, which is regulated by two independent genes: *Hd1* and *Ehd1* [19]–[22]. An association study of these three major flowering genes in the *japonica* subspecies revealed that the variations in Hd1 protein, *Hd3a* promoters, and *Ehd1* expression levels all contribute to the diversity of HD [23].

Another important yield component is plant height (PH). Plant architecture, including PH, has been subjected to strong selection throughout the domestication of rice. As a result, grain yield has been significantly increased by growing semi-dwarf varieties, which enhances absorbance of sunlight and provided stronger resistance to lodging [24].

*Ghd7* has pleiotropic effects on three agronomic traits (PH, HD, and spikelets per panicle [SPP]) [25]. *Ghd7* delays HD, increases PH and panicle size, and results in enhanced gene expression of *Ehd1* and *Hd3a* under long-day conditions. Expression pattern analysis suggested that *Ghd7* may function upstream of *Ehd1* and *Hd3a* in the rice flowering pathway. Association analysis of 19 rice cultivars identified five allelic variants of *Ghd7*. The *Ghd7* alleles with strong genetic effects were shown to increase grain yield by adapting to the long growing season of tropical regions and the *Ghd7* alleles with no or reduced effect found in temperate regions shortened the rice life cycle to ensure seed setting [25].

Candidate gene-based association mapping takes advantage of historical and evolutionary recombination events in a natural population to resolve complex trait variation to individual nucleotides [26]. Moreover, for a pleiotropic gene, association mapping can also dissect the trait correlations at the gene level because different polymorphic sites can be independently associated with different traits [27]. For example, the maize pleiotropic gene *Dwarf8*, which affects both flowering time and PH, was shown to contain two SNPs that are independently associated with the two related traits [28]. The pleiotropic gene *Ghd7* is an important gene that has been widely used in traditional breeding and is also a good target in molecular breeding. In this study, we sequenced a germplasm collection of 104 accessions of cultivated rice (*O. sativa*) and 3 common wild rice varieties (*O. rufipogon*) to identify the diverse alleles/haplotypes and key SNPs in *Ghd7* affecting PH, HD, SPP phenotypes. *Indica* and *japonica* subspecies showed two independent evolutionary processes of *Ghd7*. In addition to the point mutation causing a premature stop codon in *Ghd7* that caused a reduction in all three assayed traits, two more mutations were detected, both of which have contributed independently to rice genetic improvement.

## Results

### Population Structure

**Figure 1:**
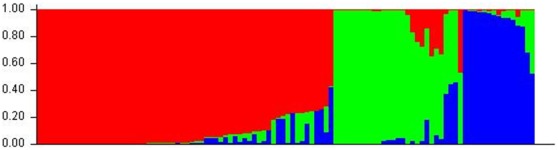
Population structure for 104 accessions. Three colors indicate the populations; red, green, and blue indicate the subpopulations 1, 2, and 3, respectively. Every accession is represented by a single vertical line with the lengths proportional to each of the subpopulations. The figure is created by STRUCTURE.

24 SSR markers were randomly selected from one each short and long arms of the 12 rice chromosomes, all of them were shown to be polymorphic among the 104 rice accessions. Individual SSR markers contained between 2–11 alleles with an average of 4.4 alleles for each marker. A significant population structure identified in the germplasm collection can be classified into three subpopulations because the highest log likelihood scores of the population structure were observed when the number of populations was set at 3 (*K* = 3; Figure 1). The first subpopulation (subpopulation 1) contained 53 accessions and was represented by 83% of the *indica* varieties; the other two subpopulations (subpopulations 2 and 3) contained 51 accessions and were represented by 88% of the *japonica* varieties (Table S1). Thus, we defined subpopulation 1 as the *indica* subpopulation and subpopulations 2 and 3 were categorized as the *japonica* subpopulation. Moreover, within the two *japonica* subpopulations, Lemont, a variety that was proven to be a *tropical japonica* variety [29], and Nipponbare, a classic *temperate japonica* variety, were distributed between subpopulations 2 and 3. Thus, the two *japonica* subpopulations may correspond with a deeper population structure division (*tropical japonica* and *temperate japonica*).

### Nucleotide Diversity and LD of *Ghd7*

The whole genomic DNA sequence of *Ghd7* from the 104 cultivars was sequenced and analyzed for its nucleotide diversity. In total, 76 SNPs and 6 insertions and deletions (InDels) were detected in the aligned 3923 basepairs (Figure S1). Across the *Ghd7* gene, 6 SNPs for every kilobase (π = 0.00621, Table 1) were found between two randomly sampled accessions in this population. Varied DNA polymorphisms were observed in different regions of the *Ghd7* genome (Table 1). In the whole germplasm population, the pairwise nucleotide diversity parameter (π) and the level of the Watterson estimator (θ_w_) in the promoter were 2- to 3-fold higher than that in the other regions. Tajima’s *D* values reached a significant positive level in the entire *Ghd7* genomic region, including the promoter (*P*<0.05). Considering the strong population stratification, we also tested these parameters within the two subpopulations (*indica* and *japonica*). The values of the π and θ_w_ in the promoter were also 2- to 3-fold higher than that in the other regions, but Tajima’s *D* values showed a negative value and reached a significant level both in the promoter and the whole gene region of *japonica* subpopulation. LD was detected in the whole genomic region of *Ghd7*, and no LD decay was observed within the whole genome (Figure S2).

**Table 1:** Summary of DNA polymorphic sites of *Ghd7* genome.

Parameter	Entire region	Promoter	5′UTR	Exon 1	Intron	Exon 2	3′UTR	
Length, bp	3923	1263	210	444	1646	330	30	
SNP sites	76	45	0	8	19	4	0	
InDels	6	4	0	0	2	0	0	
Whole population								
Π	0.0062	0.0124	–	0.0039	0.0035	0.0038	–	
θ	0.0037	0.0069	–	0.0035	0.0022	0.0023	–	
Tajima’s *D*	2.1730*	2.5443*	–	0.2990	1.6448	1.2658	–	
*indica* group								
Π	0.0028	0.0050	–	0.0027	0.0015	0.0030	–	
θ	0.0036	0.0068	–	0.0034	0.0019	0.0026	–	
Tajima’s *D*	−0.7306	−0.8374	–	−0.4747	−0.6505	0.3128	–	
*japonica* group								
Π	0.0013	0.0029	–	0.0006	0.0006	0.0004		–
θ	0.0033	0.0066	–	0.0016	0.0021	0.0014		–
Tajima’s *D*	−2.1742*	−1.9697*	–	−1.2926	−2.2250**	−1.3005		–

π, average number of nucleotide differences per site between two sequences; θ, Watterson estimator; Tajima’s *D*, test for neutral selection.

* Significant at *P*<0.05;

** significant at *P*<0.01.

### Comparison of Sequences and Haplotype Analysis

**Figure 2:**
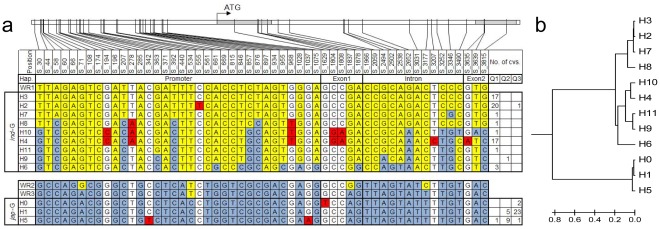
Haplotype analysis of the *Ghd7* gene region in the 104 cultivars. (a) The *Ghd7* containing two exons (indicated in gray) and the entire length of the 3923-bp genome is shown in graphics on the top. The position of every SNP is shown in the first row (SNP frequency>5%). Twelve haplotypes (H0–H11) were detected in the 104 cultivars of *O. sativa*, which can be divided into an *indica* group (*ind-G*) and a *japonica* group (*jap-G*) based on the population structure analysis. The number of cultivars (cvs) in every subpopulation is shown in the right columns: Q1 indicates the *indica* population and Q2 and Q3 indicate the *japonica* population. Yellow represents polymorphisms characteristic of the *indica* haplogroup, light blue shows the *japonica* haplogroup polymorphisms. Red indicates the new mutation. WR1–3 indicates the three wild rice varieties of *O.rufipogon.* (b) Phylogenetic tree of the twelve haplotypes (H0–H11).

The analyzed 104 accessions contained 16 haplotypes according to the detected 76 SNPs and 6 InDels (H0, H1–H15 in Figure S1). Accessions Hejiang 19 and Mudanjiang 8 were defined as haplotype H0 since both contained one premature stop codon in the first exon of *Ghd7*. 53 SNPs with a bi-allele frequency of >5% were considered for haplotype analysis. Eleven haplotypes (H1–H11) were constructed from the remaining 102 *O. sativa* cultivars (regardless of rare SNP site, H12 belongs to H2, H13–H15 belong to H1). Haplotypes H2–H4 and H6–H11 were represented mainly by accessions from the *indica* subpopulation (subpopulation 1) and hence were placed into the *indica* haplogroup. Haplotypes H1 and H5, together with haplotype H0, mainly contained accessions from *japonica* subpopulations (subpopulations 2 and 3) and were categorized into the *japonica* haplogroup (Figure 2a). In addition, two clades were detected through a phylogenetic tree analysis (Figure 2b), which also corresponded to the *indica*-*japonica* haplogroup division.

*Ghd7* showed low nucleotide diversity within the *japonica* haplogroup. The *japonica* haplogroup contained only three haplotypes (H0, H1, and H5) which were defined by three SNPs at positions S_363, S_1075, and S_1629 (indicated in red in Figure 2a). However, the *indica* haplogroup showed various haplotypes of *Ghd7*, in which haplotype H3 showed completely different SNP alleles (indicated in yellow in Figure 2a) from the *japonica* haplogroup (indicated in light blue in Figure 2a). In addition, haplotypes H4 and H6–H11 from the *indica* haplogroup partly contained *japonica* SNP alleles, and eight new mutation sites (indicated in red) were noted and accumulated in these haplotypes.

Moreover, the *Ghd7* alleles from the three wild rice varieties (*O. rufipogon*) were sequenced. WR1 from Myanmar contained completely identical SNP alleles with haplotype H3 from the *indica* haplogroup. WR2 and WR3 from Taiwan and Dongxiang (Jiangxi Province of China) carried alleles that have four and one SNPs respectively with the haplotype H1 from the *japonica* haplogroup in sequence.

### Ghd7 Protein Diversity

**Figure 3:**
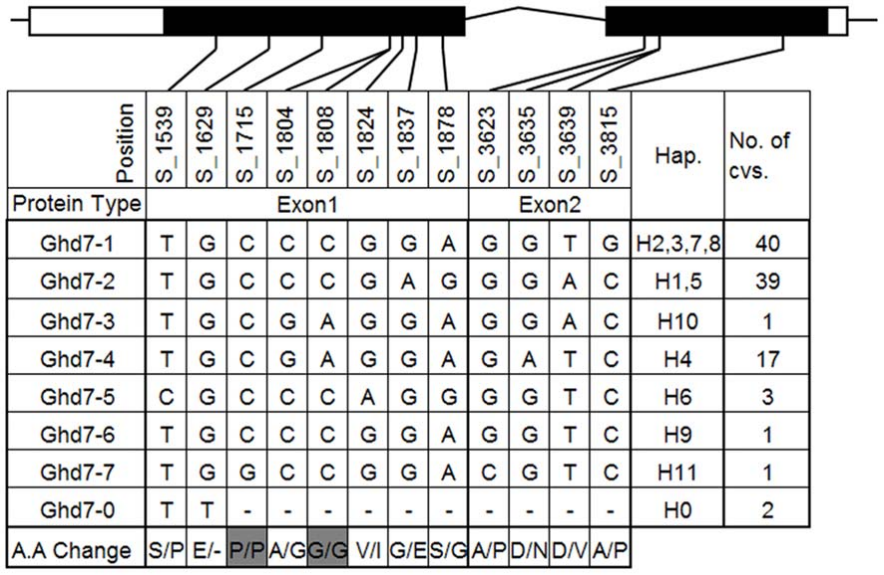
Protein diversity of Ghd7. The two exons (indicated in black rectangle) and the 5′ and 3′ UTRs (indicated in white rectangle) of Ghd7 are shown in graphics on the top. The first row indicates the position of the SNPs, the last row reveals the amino acid change. Gray indicates synonymous SNP. Eight types of Ghd7 protein were identified. Ghd7-0 was a permutation type. Hap indicates the haplotypes that share the same protein type. The numbers in the right column are the numbers of cultivars (cvs) represented in every protein type.

Considering that the nucleotide diversity in the coding region cannot exactly represent the protein diversity owing to synonymous SNPs in exons, Ghd7 protein diversity was analyzed in the present study (Figure 3). Eight protein types were identified in this population; nine non-synonymous SNPs (indicated in white), two synonymous SNPs (indicated in gray), and one premature stop codon were detected in the coding region. Haplotypes H2, H3, H7, and H8 shared the same protein type (number of accessions: N = 40), equivalent to the Ghd7-1 type in the previous study [25], the *japonica* haplogroup (haplotypes H1 and H5) shared the same protein type with Ghd7-2 (N = 39), and haplotype H10 (1 accession: Teqing) encoded the Ghd7-3 protein type. In addition, the two accessions with a premature stop codon (Hejiang19 and Mudanjiang8) contained protein type Ghd7-0. Besides the four protein types reported previously by Xue *et al*. [25], four new protein types: Ghd7-4 (N = 17), Ghd7-5 (N = 3), Ghd7-6 (1 accession: MOLOK), and Ghd7-7 (1 accession: Shufeng101) were found in this study corresponding to haplotypes H4, H6, H9 and H11, respectively. We compared the functions of the 4 major Ghd7 protein types (Ghd7-0, Ghd7-1, Ghd7-2 and Ghd7-4) on the three target traits (Table 2). Significant differences in all the three target traits were detected between Ghd7-0 and the other three types both in LD and SD. As compared to Ghd7-2 and Ghd7-3, Ghd7-4 showed earlier heading and larger SPP in 2010. The result revealed the protein diversity of Ghd7 is critical for the variation of HD and SPP in LD conditions.

**Table 2:** Comparison of means of three traits among the major 4 protein types.

	Ghd7-0		Ghd7-1		Ghd7-2		Ghd7-4			
	Means±SD	N	Means±SD	N	Means±SD	N	Means±SD	N	*F* ratio	*P*
PH(2007LD)	77.7±10.1a	2	130.4±31.8b	40	121.8±29.6b	39	125.5±27.3b	17	2.2243	0.0904
HD(2007LD)	48.0±1.9a	2	89.6±13.9b	40	86.5±11.5b	39	81.4±3.9b	17	9.1804	0.0000
SPP(2007LD)	49.2±17.4a	2	162.7±55.4b	39	146.2±44.7b	33	200.0±31.3b	10	6.5769	0.0005
										
PH(2010LD)	−	−	118.8±32.7a	31	119.5±33.0a	31	111.9±26.9a	17	0.3501	0.7057
HD(2010LD)	−	−	83.6±14.4a	31	84.7±14.0a	31	75.4±3.9b	17	3.2020	0.0462
SPP(2010LD)	−	−	173.3±48.9a	25	143.8±42.2b	23	189.5±42.3a	17	5.4496	0.0066
										
PH(2010SD)	73.5±8.6a	2	112.5±27.3b	38	114.1±29.9b	36	114.3±31.0b	17	1.2695	0.2897
HD(2010SD)	57.1±1.9a	2	83.8±9.2b	38	80.7±9.4b	36	79.9±3.7b	17	6.6881	0.0004
SPP(2010SD)	55.5±11.3a	2	157.3±45.7b	28	139.6±57.8b	33	178.3±26.6b	16	5.1143	0.0028

The first line indicates the main 4 protein types. SD, standard deviation; N, number of cultivars tested. Means followed by different letters each row are significantly different at *P* = 0.05 within one environment. *F* ratio and probability based on one-way analysis of variance.

### Association between SNPs and Traits

Taking the population structure data as covariates (Table S1), we used GLM to identify SNP–trait associations separately in the three planting tests. The varieties included in haplotypes H1–H5 were analyzed. Other haplotypes were excluded because of limited accession numbers (No. of accessions  = 1∼3). A significantly associated SNP (S_555) was detected with PH in all three planting tests (Table 3), and it was also associated with SPP in the long-day conditions of the 2007 planting test. SNP (S_555) is a C/T mutation located at 918 bp upstream of ATG. The T allele was found only in haplotype H2 and it was the only difference between haplotypes H2 and H3 (Figure 2a). Moreover, seven other SNPs (S_194, S_278, S_968, S_1804, S_1808, S_3207, and S_3635) were detected relating to SPP in all three planting tests; these seven SNPs were in complete linkage and were differentially detected between H4 and the other four haplotypes. These seven SNPs were also significantly associated with HD in both long-day condition planting tests. In addition, another 10 SNPs (S_30, S_58, S_207, S_392, S_857, S_876, S_2652, S_3252, S_3346, S_3815) were significantly associated with SPP in both the 2007 long-day and 2010 short-day planting tests. The genotype at 10 SNP sites in haplotype H4 (*indica* group) was introgressed from *japonica* haplogroup (Figure 2a).

**Table 3:** Results of GLM association of SNP traits.

Trait	PH		HD		SPP	
Site	*P*	*R^2^*	*P*	*R^2^*	*P*	*R^2^*
*2007 Long-day condition*						
S_555	0.0019	0.0998	–	–	0.0235	0.0555
7 SNP in LD^a^	–	–	0.0125	0.0657	0.0178	0.0605
10 SNP in LD^b^	–	–	–	–	0.0097	0.0716
*2010 Long-day condition*						
S_555	0.0367	0.0582	–	–	–	–
7 SNP in LD	–	–	0.0329	0.0581	0.0141	0.0664
*2010 Short-day condition*						
S_555	0.0033	0.0975	–	–	–	–
7 SNP in LD	–	–	–	–	0.0068	0.0833
10 SNP in LD	–	–	–	–	0.0183	0.0639

Result of structure-based association mapping (*P*<0.05) of haplotypes H1–H5, by GLM analysis of TASSEL. *R^2^*, the total variation explained by the SNP.

a 7 SNPs in LD:S_194, S_278, S_968, S_1804, S_1808, S_3207, and S_3635. They were in complete linkage disequilibrium and gathered in haplotype H4.

b 10 SNP in LD:S_30, S_58, S_207, S_392, S_857, S_876, S_2652, S_3252, S_3346, and S_3815. They were in complete linkage disequilibrium and they were the introgressed SNPs transferred from *japonica* to *indica*.

The average PH, HD, and SPP of each haplotype in the three planting tests were compared separately within *indica* and *japonica* subpopulations to define the haplotype–trait association (Table 4). The standard deviation of the mean values of the three traits was large because of the high trait diversity in this population. The haplotype H3 showed a significantly higher PH than haplotype H2 (with a T mutation at S_555) in all three tests.

**Table 4:** Comparison of means between different haplotypes in the three traits.

Trait	PH		HD		SPP	
Hap.	Means±SD	N	Means±SD	N	Means±SD	N
*indica*						
H2(2007LD)	111.8±16.1a	21	85.9±9.4a	21	169.2±62.4ab	21
H3(2007LD)	149.2±33.8b	17	94.4±18.0b	17	147.7±43.3a	16
H4(2007LD)	125.5±27.3a	17	81.4±3.9a	17	200.0±31.3b	10
H2(2010LD)	103.0±20.5a	16	81.4±11.9ab	16	160.0±38.9a	15
H3(2010LD)	135.4±36.5b	14	85.8±17.4a	14	189.0±59.1a	9
H4(2010LD)	111.9±26.9a	17	75.4±3.9b	17	189.5±42.3a	17
H2(2010SD)	97.8±14.8a	21	83.6±6.6a	21	152.9±34.4a	15
H3(2010SD)	127.9±29.1b	15	84.2±12.7a	15	154.3±58.4a	11
H4(2010SD)	114.3±31.0ab	17	80.0±3.7a	17	178.3±26.6a	16
*japonica*						
H1(2007LD)	122.9±32.4a	28	88.3±12.8a	28	143.8±51.4a	23
H5(2007LD)	119.1±21.9a	11	81.9±5.2a	11	151.7±24.7a	10
H1(2010LD)	120.5±37.5a	22	86.8±15.6a	22	128.0±31.4a	14
H5(2010LD)	117.0±19.6a	9	79.3±7.6a	9	168.4±46.6b	9
H1(2010SD)	112.5±33.0a	26	79.9±10.6a	26	141.3±65.6a	24
H5(2010SD)	118.2±20.5a	10	82.8±5.0a	10	135.1±31.1a	9

Hap, haplotype; SD, standard deviation; N, number of cultivars tested. Within an environment, means followed by different letters are significantly different at *P* = 0.05.

### The Association SNP S_555 was Related to the Gene Expression Level

Considering that the associated SNP S_555 was located in the promoter of *Ghd7*, the gene expression levels of 81 varieties included in haplotypes H1–H5 were measured to identify its relationship to the three investigated traits. Of haplotypes H2 and H3, which shared the same Ghd7-1 protein type, haplotype H2 (T allele at SNP S_555) showed a significantly lower expression level than that seen in haplotype H3 (C allele at SNP S_555) (Table 5). Moreover, a significant correlation between the gene expression and PH was also detected in the varieties of haplotype H2 and H3 (Table 6). However, other haplotypes (H1, H4 and H5) also had a C allele but showed a lower expression level than H3.

**Table 5:** Comparison of expression levels in H1–H5.

Hap.	Protein type	S_555	Mean ± SD	N
H2	Ghd7-1	T	1.45±1.64a	21
H3	Ghd7-1	C	2.86±1.76b	16
H4	Ghd7-4	C	1.49±0.76a	10
H1	Ghd7-2	C	1.63±0.98a	24
H5	Ghd7-2	C	1.48±0.79a	10

Hap, haplotype; SD, standard deviation; N, number of cultivars tested; characters not connected by the same letter are significantly different at *P* = 0.05.

**Table 6:** Spearman correlation analysis between expression level and three traits in three planting tests.

	PH			HD			SPP		
	N	R	*P*	N	R	*P*	N	R	*P*
2007LD	37	0.49	0.002	37	0.07	0.70	37	−0.26	0.12
2010LD	29	0.37	0.050	29	−0.07	0.74	23	−0.03	0.88
2010SD	35	0.47	0.005	35	−0.06	0.73	25	−0.25	0.23

N, number of varieties tested; R, correlation coefficient.

To further confirm the function of SNP S_555 in regulating gene expression, we compared the promoter activity of H2 and H3 by using a previously described GUS quantitative activity assay [30]. The *Ghd7* promoters of haplotypes H2 and H3 were cloned and fused with *GUS* (beta-glucuronidase) gene, and then transformed to rice callus by Agrobacterium. We compared the GUS activity of the rice positive callus. The callus carrying the *GUS* gene driven by haplotype H3 promoter showed stronger GUS activity than that of haplotype H2 (Figure S3).

### Expression Analysis of *Ehd1*

*Ehd1* was confirmed to be a downstream gene of *Ghd7* based on previous study [25]. Thus, it was used as an indicator to reflect the *Ghd7* gene activities. Ehd1 protein was reported to be functionally conserved based on the previous work [23]. An amino acid substitution in TC65 (G219R) was previously shown to decrease DNA binding activity of *Ehd1* [19] and caused late flowering in both Long-day and Short-day conditions. In order to confirm the *Ghd7* function pattern, we investigated the allele variation of *Ehd1* within the whole population. The most important amino acid substitution (G219R) did not exist in our population except for TC65 (NO.103). Thus, *Ehd1* itself does not cause large variation of phenotypes in the population. In addition, we found a 21-bp insertion in the fourth intron, which has not been previously reported. The 21-bp insertion was mainly present in the *indica* subpopulation but not in *japonica* subpopulation. This result still confirmed the conservation of *Ehd1* although 5 exceptions existed (Table S1).

The expression of *Ehd1* was evaluated throughout this population, and the expression showed a high correlation with PH and HD (Table S2). A significant correlation with *Ghd7* expression level was detected in haplotypes H2 and H3 (Table S3), in which *Ghd7* showed a high variation in expression but with the same protein type of Ghd7-1. The negative correlation between them was also consistent with the previous photoperiod study of *Ghd7* [25]. Moreover, significant difference in *Ehd1* expression was detected between H2 and H3 haplotypes (*P*<0.05). Taken together, the associated SNP_555 functions through altering *Ghd7* expression level, and further modulating *Ehd1* expression, a known downstream gene of *Ghd7*.

## Discussion

### Genetic Variation of *Ghd7*

The significant positive Tajima’s *D* parameters in the promoter and the entire genomic region of *Ghd7* suggested that the population stratification or balancing selection occurred in this locus during rice evolution and breeding. However, Tajima’s *D* parameters changed to a negative value in most of the analyzed regions when this parameter was estimated separately in the two subpopulations. The negative parameters reached a significant level in the promoter, intron, and whole region of the *japonica* subpopulation, and the negative values of Tajima’s *D* can result from positive selection. However, MOLOK in haplotype H9 and Ninghui21 in haplotype H2 are two *japonica* varieties based on a whole genome population structure analysis, but they possess *Ghd7 indica* haplotypes (see Figure 2a). When re-calculating this parameter in the *japonica* subpopulation excluding the two varieties, Tajima’s *D* becomes −0.454 and is not significant. Thereby, the negative significant Tajima’s *D* in *japonica* subpopulation probably resulted from a large amount of low frequency mutations, but not from any type of selection. Thus, more evidence is still needed before we define the selection sweep model of *Ghd7*.

Moreover, when comparing the pairwise nucleotide diversity parameter (π) with the genome-wide average level of the two subspecies (0.0016 for *indica* and 0.0006 for *japonica* of 517 landraces in China [31]), the π value of the *Ghd7* (0.0028 for *indica* and 0.0013 for *japonica*) was about twice that of the average level. This probably resulted from a wider geographical distribution of this germplasm, as it comprised varieties worldwide. In addition, the nucleotide diversity in the promoter of *Ghd7* was twice that of the coding region in both the *indica* and *japonica* subpopulations, indicating the presence of higher diversity in the promoter region of *Ghd7.* The mutations in the promoter do not cause changes to the protein leading to lower selection pressure, which probably led to accumulation of neutral mutations in the promoter region during domestication. Therefore, the high diversification of promoter provided the flexibility to adapt to various environments or to satisfy different developmental requirements. In addition, *Ghd7* is a pleiotropic gene and changes to Ghd7 protein may result in changes in the three traits (PH, HD and SPP) simultaneously. However, in many cases, cultivars having taller PH and later HD were not advantageous for rice production, but cultivars with ideal PH, proper HD, and many SPP were more desirable, which can be a result of selection of mutations in the promoter that affected *Ghd7* transcription. In accordance with this, the SNP S_555 in the promoter region was associated with the expression level of *Ghd7* and PH rather than HD and SPP. This result implied that this promoter variation had an important role in regulating the expression of *Ghd7* and PH formation.

### *Ghd7* Alleles of *indica* and *japonica* Originated from Two Distinct Ancestral Gene Pools

Comparing the *Ghd7* sequences from the 104 varieties (*O. sativa*) to the three wild rice varieties (*O. rufipogon*), haplotype H3 from the *indica* haplogroup showed an identical allele to the wild variety WR1, which suggested that it might be the original *indica* haplotype. In addition, haplotype H1 from the *japonica* haplogroup had close similarity to WR2 and WR3. As with H3 and the *indica* situation, H1 is possibly the original *japonica* haplotype. Moreover, these two possibly original gene haplotypes (H3 and H1) carried completely different alleles in the 43 SNP sites (indicated in yellow and blue, respectively, in Figure 2a). These results implied that *Ghd7* alleles in *indica* and *japonica* independently originated from two distinct ancestors, which is a result consistent with the previous conclusions that the two subspecies of rice (*japonica* and *indica*) were domesticated from two distinct ancestor gene pools [5]. In addition, the average expression levels of *Ghd7* also showed a significant difference between the two original haplotypes H1 and H3. The *indica* original haplotype, H3, showed significantly higher *Ghd7* expression than the original *japonica* haplotype, H1 (Table 5), which suggested diversity in gene expression levels existed also in the two distinct gene pools. These results indicated that the divergence of the *indica* and *japonica* subspecies predated rice domestication. However, a continuous and distinct introgression between the two subspecies was observed in the *indica* and *japonica* subspecies, suggesting *Ghd7* has undergone repeated selection over the long history of domestication. Moreover, two varieties (haplotype H9: MOLOK; haplotype H2: Ninghui21), which belonged to the *japonica* subpopulation according to the whole genome population structure analysis, had a *Ghd7* allele of *indica* type, and vice versa (haplotype H5: Dular) (Figure 2a). This may have happened via a chromosome fragment introgression between the two subspecies, as it did in the rice pericarp color-deciding gene *Rc* [12].

### Association between the Three Traits and the SNP Alleles of *Ghd7*

The SNP S_555 (C/T) significantly decreased the *Ghd7* gene expression level in haplotypes H2 and H3. SNP S_555 further altered the expression of *Ehd1* gene, which was reported to be regulated downstream of *Ghd7*. GUS activity assay experiment also revealed its function in gene expression regulation. Moreover, we found that this C/T mutation made a *cis*-element YACT change from CACT to CATT using the PLACE programs (http://www.dna.affrc.go.jp/PLACE/). This tetranucleotide (CACT) is a key component of mesophyll expression module 1; its mutation can significantly decrease promoter activity based on a previous study [32]. However, the remaining haplotypes (H1, H4, and H5) except H3 with a C allele at S_555, showed a lower expression level similar to H2, suggesting that the tetra nucleotide (CACT) was not the unique *cis*-element regulating *Ghd7* expression. In addition, in haplotypes H2 and H3, the expression level of *Ghd7* was only correlated to PH (Table 6), suggesting that of the three traits simultaneously controlled by *Ghd7*, PH is more sensitive to the expression of *Ghd7*. However, it is noteworthy that the expression levels of *Ghd7* were not the unique factors related to trait performance because different haplotypes encoded different Ghd7 proteins with distinct functions. Of these, haplotypes H2 and H3 shared the Ghd7-1 protein type, H1 and H5 shared the Ghd7-2 protein type, and H4 encoded the specific Ghd7-4 protein type (Figure 3). Hence, the diversity of Ghd7 protein was the key factor to regulate phenotype variation, and the expression level of *Ghd7* could also contribute to phenotypic diversity. This implied that different functional alleles of *Ghd7* probably contribute to phenotypic diversity by having varied effects on PH, HD and SPP.

The seven SNPs that associated with SPP in all three planting tests were present only in haplotype H4 of the five major haplotypes tested (*indica* haplotypes H2, H3, and H4; *japonica* haplotypes H1 and H5). The associations between SNP S_555 and PH, the seven complete linked SNPs and SPP in this study were present regardless of photoperiod. However, these seven mutations were associated to HD only in long-day conditions, indicating that HD is more sensitive to the photoperiod. These results were easily understandable because *Ghd7* has enhanced function under long-day conditions [25]. We also checked the expression level of *Ehd1* for varieties within haplotype H4. Throughout the whole population, the expression level of *Ehd1* in H4 was the lowest as compared to other haplotypes. Moreover, no correlation was detected between expression level of *Ehd1* and HD. Thus, it is speculated that *Ehd1* expression cannot reflect the function of the 7 SNP in haplotype H4, the special Ghd7 protein of H4 together with its expression level can regulate related traits in a unique pathway. More work would be needed to answer how the 7 SNPs contribute to these trait performances.

In addition, 10 other SNPs were associated to SPP in both the 2007 long-day and 2010 short-day planting tests. The 10 SNPs were introgression alleles between *japonica* and *indica* haplogroup, such as haplotype H4 and H1 (Figure 2a). It is understandable that the structure-based association analysis cannot sufficiently distinguish the true associations of those alleles that were differentially related among subgroups because their distributions coincided with population structure [33], [34]. Thus, more evidence is needed to confirm this association using a large natural population representing a wider genetic resource.

### New Strategies of *Ghd7* for Rice Breeding

Besides the previously reported premature stop mutation in *japonica* subspecies that leads to a reduction in all three traits [25], two other kinds of mutations associated with PH, HD, and SPP were identified. These three kinds of mutations functioned separately for rice adaptation and breeding. For example, in the *indica* subspecies, the mutation of C to T at SNP S_555 reduced the gene expression level and decreased PH; this variation allowed the plant to be more resistant to lodging without an influence on HD and yield. On the other hand, haplotype H4 carried the seven completely linked association mutations; among all the investigated accessions, 17 possessed haplotype H4, including many typical high-yield varieties widely grown in South and East China, such as Nanjing11, Guichao 2, Fengaizhan, and Huanghuazhan. This indicated the favorable allele (H4) was well utilized in developing rice of high yielding variety. In addition, Teqing (H10), a variety widely cultivated in South China in the 1980s, carried a strong allele of *Ghd7* [25], which also showed similar SNP alleles to haplotype H4. However, in the case of *japonica* subspecies, when the planting area was extended into temperate zones (northern regions), a significant shortage of HD was required for plants to set seeds. Thus, only the previously reported premature stop mutation of *Ghd7* in *japonica* subspecies [25], which creates the rice photoperiod insensitivity, can complete its life cycle in a short summer period.

The natural variations of *Ghd7* contribute greatly to rice adaptation and genetic improvement. These three kinds of mutations provided us a theoretical clue to the flexible use of this pleiotropic gene *Ghd7* in modern molecular breeding [27]. Specific markers could be developed for selection of the favorable haplotypes to meet the demand for varieties in different ecotypes.

## Materials and Methods

### Plant Materials and Phenotypic Data Collection

A total of 104 accessions of *O. sativa* comprising 59 *indica*, 43 *japonica*, and 2 accessions with admixed genetic background were used. Most accessions are landraces, but some correspond to modern cultivars. An additional three common wild rice varieties (*O.rufipogon*) were from IRRI (International Rice Germplasm Collection). The basic information for each germplasm appears in Table S1. The plants were grown three times on a bird net-equipped field on the experimental farm of Huazhong Agricultural University, Wuhan, China. No specific permits were required for the described field studies, and the field studies did not involve endangered or protected species. Planting dates were 19 May 2007, 19 May 2010, and 25 June 2010. Plants sowed on 19 May grew mostly under long-day conditions, whereas those sowed on 25 June were mostly under short-day conditions. Ten plants were transplanted in a single row with 16.5 cm between plants and 26.4 cm between rows for every accession. Field management was performed according to normal agricultural practices. HD was defined as the days from sowing to the appearance of the first panicle. SPP was measured as the total number of spikelets per plant divided by its panicle number. PH was measured from the surface of the ground to the tip of the tallest panicle in the plant. Except for two marginal plants in each side, eight independent plants were used to score the three phenotypic data sets.

### DNA Extraction, PCR, and Sequencing

Fresh leaves were harvested from field-grown plants and genomic DNA was extracted using the cetyl-trimethyl ammonium bromide method [35]. Genomic DNA including 1263-bp promoter regions, 210-bp 5′ UTR, 774-bp coding region, 1646-bp intron, and 30-bp 3′ UTR were amplified from genomic DNA using LA *Taq* (Takara). Table S4 provides a list of all primers used for polymerase chain reactions (PCRs) and sequencing. PCRs were conducted using standard PCR protocols with 2×GC buffer I (Takara). For sequencing, 5 µL PCR product was digested with 5 U EXOI (Biolabs) and 0.13 U Shrimp Alkaline Phosphatase (Takara) together with 1×PCR buffer and incubated at 37°C for 1 h; the reaction was stopped by maintaining the PCR product in 80°C for 20 min. To ensure accuracy, sequencing was independently performed three times in both forward and reverse primers on ABI 3730 with BigDye terminator sequencing kits (Applied Biosystems). Sequence contigs were assembled by SEQUENCHER 4.1.2 (Gene Codes Corporation). Sequences of the 12 haplotypes of *Ghd7* can be found in the GenBank/EMBL data libraries with accession codes of JF926532–JF926543.

### Gene Expression Analysis and Quantitative GUS Activity Assay

Leaves from three to five independent plants of each accession were harvested 22d after germination, when they were in the vegetative growth period under long-day conditions, to minimize the difference in developmental stage among accessions. Total RNA was extracted using TRIzol (Invitrogen). Total RNA (2 µg) was reverse-transcribed using SuperScriptII reverse transcriptase (Invitrogen) in a final volume of 20 µL to obtain cDNA. Real-time PCR was performed using gene-specific primers in a total volume of 25 µL with 2 µL of the reverse-transcribed product, 0.25 mM gene-specific primers, and 12.5 µL SYBR® Premix Ex *Taq*™ (Takara) on a 7500 real-time PCR system (Applied Biosystems) according to the manufacturer’s instructions. Four technical replicates were performed for each sample. The rice *Actin* gene was used as the internal control. The expression level data were obtained using the relative quantification method. Table S4 lists the primers used for this analysis.

The promoters of H2 and H3 haplotype *Ghd7* were isolated and fused with *GUS* (beta-glucuronidase) gene, respectively. The constructs were transformed independently to ZhongHua 11, a *japonica* variety. After 3 times selection with Hygromycin, the positive callus was used for GUS activity evaluation. The method for quantitative GUS activity assay was followed a previous work [30].

### Population Structure Analysis

Twenty-four simple sequence repeat (SSR) markers, one each in the short and long arms of the 12 rice chromosomes, were randomly selected for genotyping the 104 ricevarieties according to the genetic map developed by Temnykh *et al.* [36]. The 24 markers were RM529, RM522, RM526, RM211, RM411, RM60, RM518, RM348, RM574, RM274, RM508, RM412, RM427, RM172, RM339, RM408, RM553, RM321, RM484, RM239, RM224, RM479, RM247, and RM463. PCR was performed as described above and PCR products were separated on 4% polyacrylamide denaturing gels to determine the alleles of each marker. Program STRUCTURE 2.3.2 [37] was used to infer population structure using a burn-in of 10,000, a run length of 100,000, and a model allowing for admixture and correlated allele frequencies. The number of subpopulations *K* from two to five was tested and five independent runs yielded consistent likelihoods of the population structure for each *K*. The most probable structure number of *K* was calculated based on Evanno *et al.* [38] using an ad hoc statistic Δ*K* based on the rate of change in the log probability of data between successive *K* values.

### Statistical Analysis

The genomic sequences and protein sequences were aligned by ClustalW 2.0.9, and the alignments were used as an input format into TASSEL [39]. Nucleotide diversity and Tajima’s *D* statistics were calculated using the DnaSP 5.0 program [40]. Linkage disequilibrium (LD) was estimated by using standardized disequilibrium coefficients (*D*′) and squared allele-frequency correlations (*r^2^*) for pairs of SNP loci according to the TASSEL program. TASSEL was also used to identify SNP–trait associations by generating a general linear model (GLM). The difference of gene expression level and the trait comparison of each haplotype were examined by ANOVA, and the Duncan multiple range test and critical test were conducted if the analyses were significant (*P*<0.05). Correlation between three traits and gene expression level was examined by the Spearman correlation coefficient test. Statistical analysis was performed by the STATISTICA software (StatSoft 1995). The evolutionary relationship among the 12 haplotypes were inferred using the UPGMA method and phylogenetic analyses were conducted in MEGA4 software [41].

## Supporting Information

Figure S1 **16 haplotypes of** ***Ghd7*** **in the 104 rice varieties.** The position of every SNP and InDels are shown in the first row (SNP frequency>1%). Two exons indicated in gray and one intron of *Ghd7* were shown in the second row. The number “0” indicates deletion. 16 haplotypes (H0–H15) were detected in the 104 cultivars of *O. sativa*, which can be divided into an *indica* group (*ind-G*) and a *japonica* group (*jap-G*) based on the population structure analysis. The number of cultivars (cvs) in every subpopulation is shown in the right columns: Q1 indicates the *indica* population, Q2 and Q3 indicate the *japonica* population. Yellow represents polymorphisms characteristic of the *indica* haplogroup, light blue shows the *japonica* haplogroup polymorphisms. Red indicates the new mutation.
(TIF)

Figure S2 **Linkage disequilibrium over the whole genomic of** ***Ghd7.***
(TIF)

Figure S3 **Relative GUS activity between the promoter of H2 and H3.**
(PDF)

Table S1 **Basic information for 104 tested rice accessions.**
(XLS)

Table S2 **Correlation coefficients between the expression levels of Ghd7/Ehd1 and the three related phenotypes in the whole population.**
(PDF)

Table S3 **Correlation coefficients between the expression levels of Ghd7/Ehd1 and the three related phenotypes within haplotypes H2 and H3.**
(PDF)

Table S4 **Primers used in this research.**
(PDF)
