# Supplementary figures and images for: Evolution and Association Analysis of Ghd7 in Rice
Source: PLoS One. 2012 May 30;7(5):e34021. doi: 10.1371/journal.pone.0034021 (PMC3364234; doi:10.1371/journal.pone.0034021)

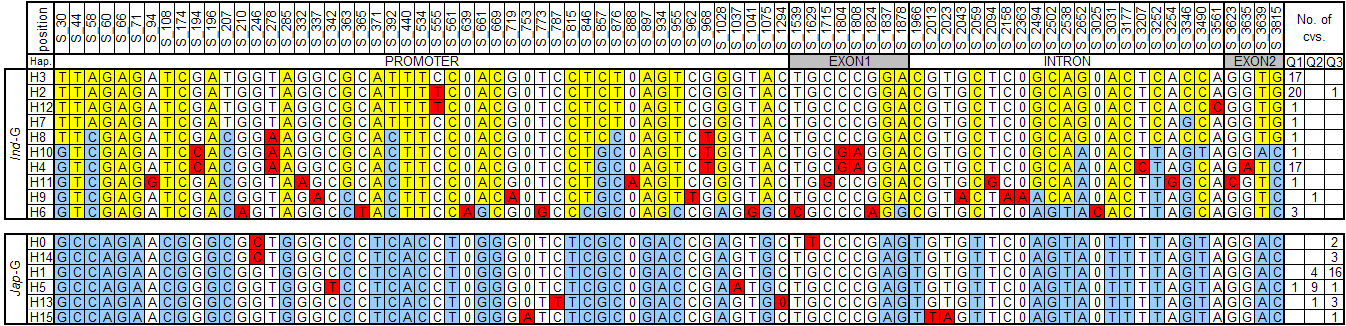

Supplement: Figure S1 — 16 haplotypes of Ghd7 in the 104 rice varieties. The position of every SNP and InDels are shown in the first row (SNP frequency>1%). Two exons indicated in gray and one intron of Ghd7 were shown in the second row. The number “0” indicates deletion. 16 haplotypes (H0–H15) were detected in the 104 cultivars of O. sativa, which can be divided into an indica group (ind-G) and a japonica group (jap-G) based on the population structure analysis. The number of cultivars (cvs) in every subpopulation is shown in the right columns: Q1 indicates the indica population, Q2 and Q3 indicate the japonica population. Yellow represents polymorphisms characteristic of the indica haplogroup, light blue shows the japonica haplogroup polymorphisms. Red indicates the new mutation. (TIF) [file pone.0034021.s001.tif]

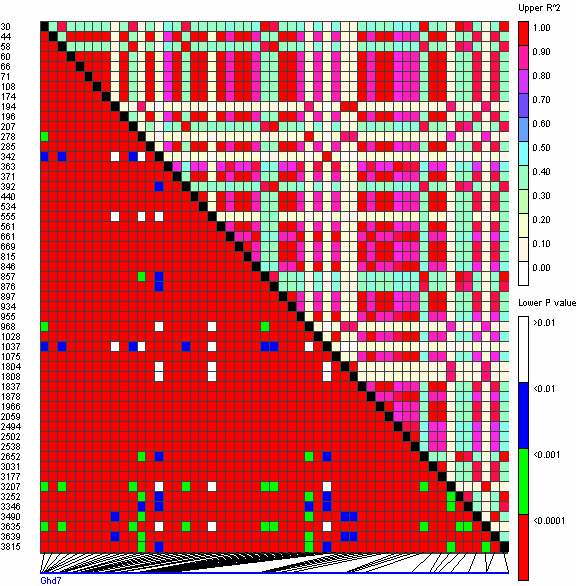

Supplement: Figure S2 — Linkage disequilibrium over the whole genomic of Ghd7. (TIF) [file pone.0034021.s002.tif]
