# Supplementary material for: Evolution and Association Analysis of Ghd7 in Rice
Source: PLoS One. 2012 May 30;7(5):e34021. doi: 10.1371/journal.pone.0034021 (PMC3364234; doi:10.1371/journal.pone.0034021)
Supplement: Figure S3 — Relative GUS activity between the promoter of H2 and H3. (PDF) [file pone.0034021.s003.pdf]

Figure S3: Relative GUS activity between the promoter of H2 and H3.

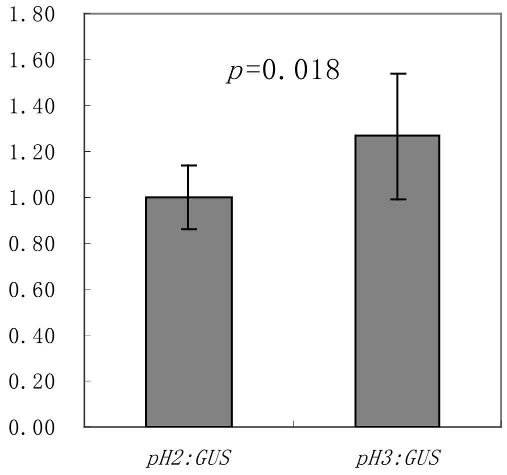

The *p*-value was calculated by two-tailed student's *t* test, N=30.
