# Supplementary material for: Evolution and Association Analysis of Ghd7 in Rice
Source: PLoS One. 2012 May 30;7(5):e34021. doi: 10.1371/journal.pone.0034021 (PMC3364234; doi:10.1371/journal.pone.0034021)
Supplement: Table S2 — Correlation coefficients between the expression levels of Ghd7/Ehd1 and the three related phenotypes in the whole population. (PDF) [file pone.0034021.s005.pdf]

Table S2: Correlation coefficients between the expression levels of *Ghd7/Ehd1* and the three related phenotypes in the whole population in long day condition 2007

	Ghd7	*Ehd1*	PH	HD
Ehd1	-0.185			
PH	0.339	-0.43**		
HD	0.061	-0.49**	0.451**	
SPP	-0.225	0.077	-0.132	-0.12

*, ** indicate P<0.01 and P<0.001 respectively.
