# Supplementary material for: Evolution and Association Analysis of Ghd7 in Rice
Source: PLoS One. 2012 May 30;7(5):e34021. doi: 10.1371/journal.pone.0034021 (PMC3364234; doi:10.1371/journal.pone.0034021)
Supplement: Table S3 — Correlation coefficients between the expression levels of Ghd7/Ehd1 and the three related phenotypes within haplotypes H2 and H3. (PDF) [file pone.0034021.s006.pdf]

Table S3: Correlation coefficients between the expression levels of *Ghd7/Ehd1* and the three related phenotypes within haplotypes H2 and H3 in long day 2007

	Ghd7	*Ehd1*	PH	HD
Ehd1	-0.503*			
PH	0.635**	-0.559*		
HD	0.16	-0.516*	0.502*	
SPP	-0.358	0.303	-0.296	-0.264

*, ** indicate P<0.01 and P<0.001 respectively.
