# Supplementary material for: Evolution and Association Analysis of Ghd7 in Rice
Source: PLoS One. 2012 May 30;7(5):e34021. doi: 10.1371/journal.pone.0034021 (PMC3364234; doi:10.1371/journal.pone.0034021)
Supplement: Table S4 — Primers used in this research. (PDF) [file pone.0034021.s007.pdf]

Table S4: Primers used in this research

Name	Forward primer	Reverse primer
Full	caacttgccctgtcttcttcttc	tagcgcaggatcagtcatatatagttagtg
Sequence 1-2	gcaaggggatgtctaaacga	aatttttgaccgtcggattc
Sequence 3-4	caagctggtgggatcgag	ttgccgaagaactggaactc
Sequence 5-6	ttatccgttcatgtcgatgg	accgaactcgaaaagcacac
Sequence 7-8	catacggatccagcctctgt	ttgcaatgatgcgtattcac
Sequence 9	gtcatattgtgggagcacgt	
Sequence 10		accatctccttgggcatcga
Sequence 11	tagaactgcaaggagatgca	
Ghd7-RT	aggtgctacgagaagcaaatcc	gggcctcatctcggcatag
actin-RT	tgctatgtacgtcgccatccag	aatgagtaaccacgctccgtca
Ghd7proF/R	aagcttgtgcgggtgagacacctgcacgtg	ctcgaggaacggataaatcaaactcgatcg
